# Do Aging and Tactile Noise Stimulation Affect Responses to Support Surface Translations in Healthy Adults?

**DOI:** 10.1155/2016/2941964

**Published:** 2016-04-19

**Authors:** Marius Dettmer, Amir Pourmoghaddam, Beom-Chan Lee, Charles S. Layne

**Affiliations:** ^1^Memorial Bone & Joint Research Foundation, 1140 Business Center Drive Suite 101, Houston, TX 77043, USA; ^2^Health and Human Performance Department, University of Houston, 3855 Holman Street, Houston, TX 77204-6015, USA

## Abstract

Appropriate neuromuscular responses to support surface perturbations are crucial to prevent falls, but aging-related anatomical and physiological changes affect the appropriateness and efficiency of such responses. Low-level noise application to sensory receptors has shown to be effective for postural improvement in a variety of different balance tasks, but it is unknown whether this intervention may have value for improvement of corrective postural responses. Ten healthy younger and ten healthy older adults were exposed to sudden backward translations of the support surface. Low-level noise (mechanical vibration) to the foot soles was added during random trials and temporal (response latency) and spatial characteristics (maximum center-of-pressure excursion and anterior-posterior path length) of postural responses were assessed. Mixed-model ANOVA was applied for analysis of postural response differences based on age and vibration condition. Age affected postural response characteristics, but older adults were well able to maintain balance when exposed to a postural perturbation. Low-level noise application did not affect any postural outcomes. Healthy aging affects some specific measures of postural stability, and in high-functioning older individuals, a low-level noise intervention may not be valuable. More research is needed to investigate if recurring fallers and neuropathy patients could benefit from the intervention in postural perturbation tasks.

## 1. Introduction

Maintenance of upright stance is a demanding task that requires complex neuromuscular control. However, in healthy adults, postural control mostly occurs without conscious contribution. More impressively, the central nervous system (CNS) is able to maintain or reestablish balance after experiencing postural threats by applying a number of different compensatory strategies. This remarkable capacity of the CNS to respond to a vast amount of different balance perturbations has been the topic of numerous motor control experiments. To investigate the postural control mechanisms responsible for maintenance of postural stability, researchers often employ experimental methods that involve tasks resembling real-life situations, such as postural perturbance in the form of rotational or translational movements of the support surface [1, 2]. Assessing temporal and spatial characteristics of the associated balance processes then allows investigators to make conclusions about the effects of task characteristics or effects of pathology and aging on such postural features. Additionally, individual postural response characteristics to sudden platform perturbations have shown to be a significant predictor of future falls [3].

It is well known that sensorimotor control and static balance performance decline due to both aging [4] and pathologies [5], and such deterioration is reflected by results from postural perturbation experiments [6]. Aging and task constraints significantly affect responses to postural perturbations and associated orchestration of corrective movements [7–14]. It is believed that about 50% of all falls are caused by sudden movements of the base of support, trips, and slips or because of other external perturbations affecting center of mass displacement over the base of support [15]. Whereas a healthy sensorimotor system is able to overcome perturbance with rapid, accurate muscular responses, in many older adults, this efficiency is reduced, whereas responses to perturbation may become delayed or inappropriate [16, 17].

To improve older adults' postural performance and to improve corrective responses during postural perturbations, several different interventions have been developed. Training interventions have been shown to be useful to improve neuromuscular performance and to improve balance [18]. Alternatively, it is possible to target and augment sensory feedback from peripheral sensory sources to improve sensorimotor control and postural performance.

An improvement of postural recovery has been shown when using polyethylene tubes around the plantar surface in experiments requiring participants to generate a rapid stepping response due to support surface perturbations [19, 20]. This indicates that enhancement of tactile information stemming from the mechanoreceptors of the feet could be beneficial in balance-threatening situations. Some interventions aiming at the improvement of sensory information from relevant sources for postural control apply the principles of stochastic resonance (SR).

SR is a phenomenon associated with addition of noise into a nonlinear system that is applicable to natural and some man-made systems [21–23]. SR describes the enhancement of information transmission and weak stimuli detection when noise is added to a system. In biological systems, the application of “threshold SR” is based on the lower limits of sensory signals humans can consciously perceive. A “threshold” is defined as the stimulus magnitude required to be perceived by the central nervous system. A beneficial effect in a system can be observed when combination and interaction of (a) a specific threshold, (b) a below-threshold stimulus (subthreshold stimulus), and (c) nonzero-level noise [24] cause a positive effect on system function in contrast to higher level noise, which has negative effects on system function [25].

The value of SR for postural control purposes has been shown in a number of studies [20, 26–33]. Specifically, receptors that provide pressure information on the plantar surface have been a major target for augmentation of sensory feedback [32–34] with the goal of stabilization of upright stance. More recently, mechanical stimulation has been shown to be valuable in more demanding postural tasks such as tasks including visual conflicts like a sway-referenced surrounding [35] or a rotating visual scene [36]. Despite promising results regarding the utilization of SR in specific tasks, it is yet unknown how noise induction affects postural control processing during balance-threatening tasks that require rapid reactive responses. Results from such experiments could shed light on potential benefits or limitations of vibration tools for modifying postural control and improving upright stability in situations where improved control is crucial for prevention of falls.

Here, we designed an experiment to determine the specific differences in healthy younger and older adults when facing a translational support surface perturbation. We hypothesized that spatial and temporal features of perturbation response are different in regard to age group.

We also employed a SR based intervention to investigate potential benefits from low-level noise induction. We hypothesized that subthreshold noise would affect spatial and temporal features of motor control (generation of corrective postural responses), but potentially only in older adults.

## 2. Materials and Methods

### 2.1. Participants

This study was conducted according to University of Houston policies concerning the protection of participants in human research. The protocol was approved by the University of Houston Committee for the Protection of Human Subjects (CPHS). All participants in the study signed an informed consent form before participation. We recruited 20 healthy older (*n* = 10) and younger (*n* = 10) participants. To be included in the study, prospective participants had to be free of any significant medical conditions, which included both physical and cognitive impairments. Age range for inclusion in the study was 20–35 years for the younger age group and 70–85 years for the older age group. The age range for the older adults group was based on existing knowledge about the onset of sensory decline and associated higher risk for falls. It has been shown that there is an age-related acceleration of sensory decline around the age of 70 [37]; therefore, this age was chosen as the lower limit for inclusion (see Table 1 for demographic information).

Physical health and inclusion in the study were determined based on a modified version of the Physical Activity Readiness Questionnaire (PAR-Q). Answers to the PAR-Q were analyzed to determine whether participants could be included in the study, which was only if they answered “no” to all question items (or reported health concerns that did not put them at any risk during the experimental trials and/or could have affected postural performance) and reported to be in overall good health. If they reported current use of medication, we further evaluated whether the medication would or would not interfere with postural performance. Due to the nature of the experiments, only individuals without cognitive impairments, as represented by a score of 27 or higher on the Minimental State Examination (MMSE) [38], were included in the study. An initial sensory detection test was administered to ensure that older adults displayed an elevated sensory threshold (touch/pressure threshold). Only those individuals displaying an elevated threshold (compared to the younger group) were included in the study.

Participants were excluded if they reported significant physiological problems based on question items on the modified PAR-Q. This included but was not limited to general health problems or severe sensorimotor impairments (e.g., neuropathies, chest pain upon exertion, dyspnea, infection, and functionally significant musculoskeletal dysfunction). Due to the potential effects of obesity on postural stability, individuals with a body mass index (BMI) > 30 kg/m^2^ were excluded.

### 2.2. Mechanical Vibration of the Foot Sole

**Figure 1:**
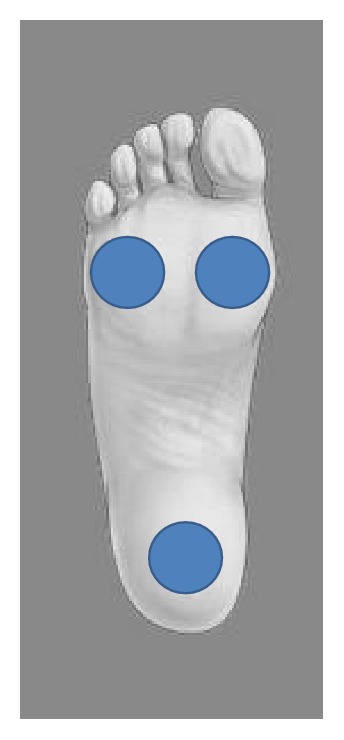
Position of tactors under the sole of the foot (1st and 5th metatarsal-phalangeal joint region and heel).

Vibrotactile stimulation of the foot sole was administered via vibrating chips embedded into a custom-made foam sole: three vibration stimulators (C-2; Engineering Acoustics, FL) were integrated into a custom-made silicone rubber sole [27] with a hardness of Shore 50A [20]. There were three specific locations for the tactors under the feet to expose cutaneous afferents to tactile stimuli (Figure 1).

The C-2 tactors that were embedded in the rubber sole are moving magnet motor devices with a diameter of 30.5 mm, a height (in actuation direction) of 7.9 mm, and maximum displacement amplitude of about 0.635 mm. The use of C-2 tactors as actuators has been established via foot sole stimulation experiments that exposed participants to low-level vibration noise [39, 40]. Other authors suggested the use of C-2 tactors based on their dimensions, the possible frequency range that is available, and the range of amplitudes [20]. All six tactors were connected to a control box including amplifiers, a memory bank for storing sequences of stimuli, and the power supply (battery). The control box was connected to a PC via a USB connector. Custom-designed software was used to generate pseudorandom white-noise vibration. For the current experiments, a white-noise signal was added to a generated sinusoidal signal band limited to 1 Hz to about 500 Hz, thereby including vibration frequencies that encompass the response bandwidth of mechanoreceptors of the foot sole.

The amplifiers for the specific tactors are based on audio amplifiers. Magnitude of vibration stimuli (gain) is expressed in terms of voltage. The drive current is approximately 300 mA (rms) at the highest gain (which is 4). The current can then be modified/reduced by manipulating gain/voltage. In addition to the gain integer, an attenuation parameter (1–63) can be set which scales the gain between global levels (1–4). Therefore, the generated amplitude is based on two parameters: the global integer (1–4) and then a number (1–63) for the attenuation between global integers. Customized software was created to allow the investigators to manipulate stimulus magnitude as required using a guided user interface displaying magnitude of stimulus as % of maximum vibration output.

### 2.3. Force Data Collection

**Figure 2:**
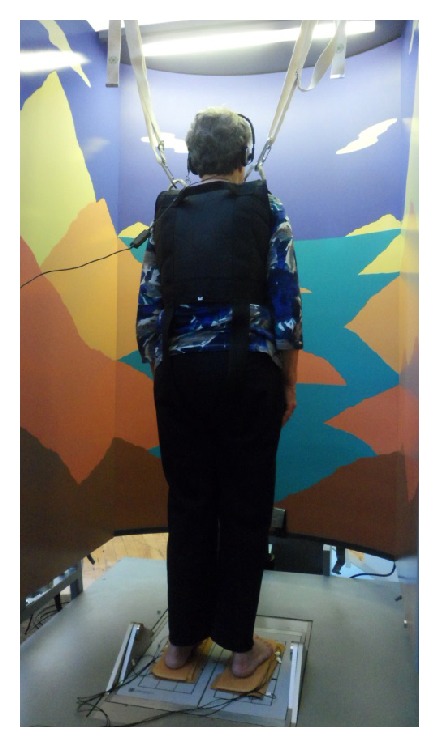
NeuroCom EquiTest system. Participants stood on customized soles with embedded vibrating units during all trials.

Center-of-pressure data was computed based on force data acquired using a force plate system (NeuroCom EquiTest, NeuroCom Intl, Clackamas, OR). The device consists of a dynamic 18′′  × 18′′ dual force plate and provides both rotation (±10° from center, either toes-up or toes-down, maximum velocity of 50°/s), and translation capabilities (±6.35 cm from center, maximum of 12.7 cm in the forward-backward direction, and maximum velocity of 20 cm/s). A visual scene can be programmed to move independently of the force plates via servomotors (±10° from center, maximum velocity of 15°/s). This allows for a type of postural investigation called computerized dynamic posturography (CDP). The NeuroCom system (Figure 2) measures forces exerted by participants' feet while providing a safety harness to prevent falls in participants (see Figure 2). The NeuroCom measurement device has been used in a wide variety of clinical and scientific studies related to postural control [41–43]. Force plate data was collected at 100 Hz and processed via Windows-based software on a connected computer (Research module, NeuroCom software version 8.0, NeuroCom Intl., Clackamas, OR).

## 3. Procedures

### 3.1. Foot Sensitivity Testing

It is known that older adults exhibit higher sensory thresholds [37, 44]. An initial test determined whether participants in this study (older group) actually had elevated tactile thresholds. At the beginning of the experimental session, individuals were asked to remove shoes and socks and to sit in a chair. We applied a tactile sensation via a Semmes-Weinstein Monofilament (5.07/10 g). A forced-choice method was applied, whereas participants were asked to report whether they could feel a stimulus or not. All testing was done over the first metatarsal joint, and four trials were performed on each foot. Older adults were included if they did not feel the stimulus in more than three out of four trials on each foot [45].

### 3.2. Stimulator Fitting and Familiarization

All participants of the study were accustomed to the rubber soles, which were custom-fitted to ensure positioning of the tactors at the foot positions described earlier (Figure 1). Participants were asked about their shoe size prior to data collection. Based on this information, the tactor sole was modified to fit the participant's foot (using silicone blocks that could be inserted in the midfoot section of the sole, to increase or decrease size). Participants were seated comfortably with their feet planted on the tactor surface. The necessity for potential adjustments was then determined via visual inspection by the principal investigator to ensure an optimal fit.

### 3.3. Sensory Threshold Evaluation

The evaluation of individual threshold was crucial in this experiment since it allowed the determination of the respective experimental stimulus magnitude/amplitude (approximately 90% of sensory threshold intensity). Stimuli set at a level of around 90% have been shown to be effective to elicit the desired effects of subthreshold vibration and have been used in previous studies that have included subthreshold vibration noise to the feet [27, 34, 39].

To determine the 90% threshold level, participants were asked to stand on the rubber sole containing the tactors. Vibration stimuli of about 5 s duration were presented at intervals of several seconds. The individual threshold was determined using a method applying stimuli at specific levels and based on feedback from each participant [46]. Participants were asked to indicate when they could feel the vibration under the sole of the foot (“forced-choice”). A large stimulus was provided (one that is detectable) followed by an undetectable one. The next stimulus magnitude was calculated based on the midpoint of the first two. If a participant was able to detect this new stimulus, the next stimulus was based on the midpoint between the former, undetected stimulus and this detected level. The final threshold was then evaluated based on a predetermined range between undetected and detected stimulus [37].

During the experimental trials, participants stood barefoot on the rubber sole containing the vibrating tactor elements. They wore a harness that was attached to a metal frame around the NeuroCom to prevent injury if a fall occurred. Participants were blinded regarding the current vibration condition.

The support surface moved (translation of support surface) in posterior direction for three consecutive times with a small displacement (familiarization trials). During these initial trials, participants were familiarized with the sensation of the translating platform. The next 10 trials were the experimental trials in blocks of five trials with stimulation either turned on or turned off (order of blocks randomized). The trials consisted of customized translations (400 ms duration) with amplitude matched to the participants' height, to elicit a sway equivalent of about 3.2 degrees. The amplitude of displacement (in inches) was calculated and generated by the NeuroCom system using the following equation:

(1) $$\begin{array}{c} 2.25*\left(\;\frac{height}{72}\;\right), \end{array}$$

which is the standard for the clinically established Motor Control Test [47]. Participants were instructed to try to stand as quiet as possible and to take a step if necessary. Participants were given a “Go” signal after which the platform translated (after a pseudorandom period between one and three seconds). After five trials, participants were given a two-minute break to prevent fatigue.

### 3.4. Data Reduction

Force plate data was collected at 100 Hz and for durations of 3 seconds (for .5 seconds before onset of perturbation and for 2.5 seconds after onset of perturbation). Outcome measures were generated either via integrated software (Research module, NeuroCom software version 8.0, NeuroCom Intl.) or with a custom MatLab script (Mathworks, USA R2013b). Anterior-posterior path length was calculated by summarizing the total anterior-posterior displacement of COP (as calculated from force plate data) for each trial. Data was then averaged over five trials.

Temporal (latency) analysis of evoked, active neuromotor responses was calculated based on the initiation of each individual's active response (force response) after onset of perturbation. The onset of force activation is based on a sudden change of the position of the center of force due to force generation at the feet. This “take-off” instant is directed forward for backward translations [48]. The measurement of latency based on force plate data has to be adjusted, since the responses measured by the force plate are lagging compared to a measurable EMG response around the ankles by about 30–50 ms [6, 49]. To determine the actual onset of an active corrective response, we used a method applied to COP data in an earlier study [50]. This included an analysis of the derivatives of the COP time-series and subsequent visual inspection of the data. This method is based on the observation of several zero crossings in the second derivative (acceleration) of COP position data, which is considered the “passive recovery” phase that does not include active generation of torque for compensation of the perturbation (but is based on passive structures). The onset of active recovery is defined as the first zero crossing of the first derivative (velocity) after this first phase [50]. A MatLab script was used to determine this instant with a visual inspection follow-up, whereas in cases where a lack of zero-crossings was present, visual inspection was used to determine initiation of active response. Results from five trials were then averaged.

Additionally, we assessed maximal COP excursion in anterior-posterior direction. COP excursion was based on the maximum value for anterior-posterior COP displacement during each trial. Initial COP position was defined as average COP position in the .5 seconds before onset of support surface translation. A custom MatLab script was used to find the point of maximum excursion throughout each trial (in relation to baseline position). Maximum anterior-posterior COP displacement was then averaged over five trials (one block) per subject, a method applied before elsewhere [51].

### 3.5. Data Analysis

Mixed-model ANOVA was utilized for statistical comparisons. There were two independent variables (age and vibration), with one between-groups factor (age) and one within-group factor (vibration). The mixed-model approach allowed the analysis of main effects (vibration and age) and potential interactions (age and vibration) to test the main hypotheses. Significance of statistical comparisons was tested at *α* < .05 level and effect size was evaluated by generating partial eta-squared (*η*_*p*_^2^).

## 4. Results

### 4.1. Vibration Threshold

Prior to the experimental data collection, a vibration threshold test was conducted with each participant to determine the vibration levels corresponding to lowest perceivable (threshold) vibration amplitude. The results are expressed as a fraction of the maximal vibration amplitude as determined by the vibration controller unit. The younger age group showed high sensitivity of the foot receptors, requiring only very low vibration amplitude and the older age group required significantly higher vibration amplitudes. Results from this initial test have been published before [35].

### 4.2. Response to Postural Perturbation

**Figure 3:**
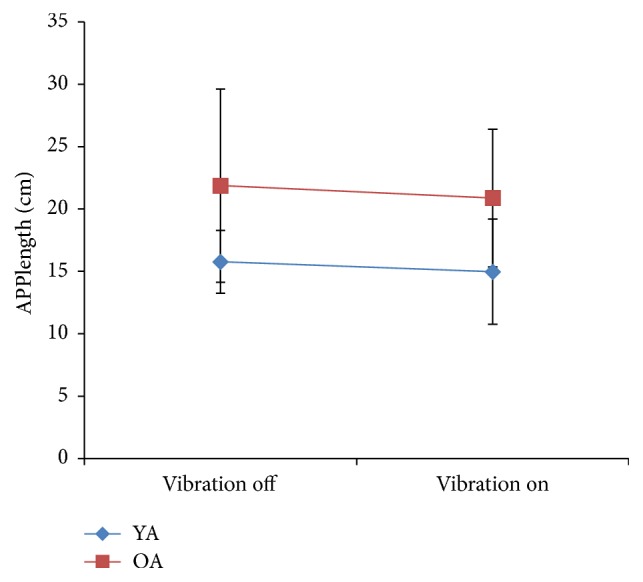
Comparison of APPlength in postural perturbation task between older adults (OA) and younger adults (YA).

Results are summarized in Table 2. APPlength was significantly greater in older adults than in younger adults (Figure 3), *F*(1, 18) = 7.482, *p* = 0.012, and *η*_*p*_^2^ = .303. Over the course of each trial (2.5 s), older adults' total COP displacement was on average larger than that in younger adults. Vibration did not affect APPlength in older or younger adults.

Maximum COP excursion did not differ between older and younger adults, and there was no effect of vibration on COP excursion.

**Figure 4:**
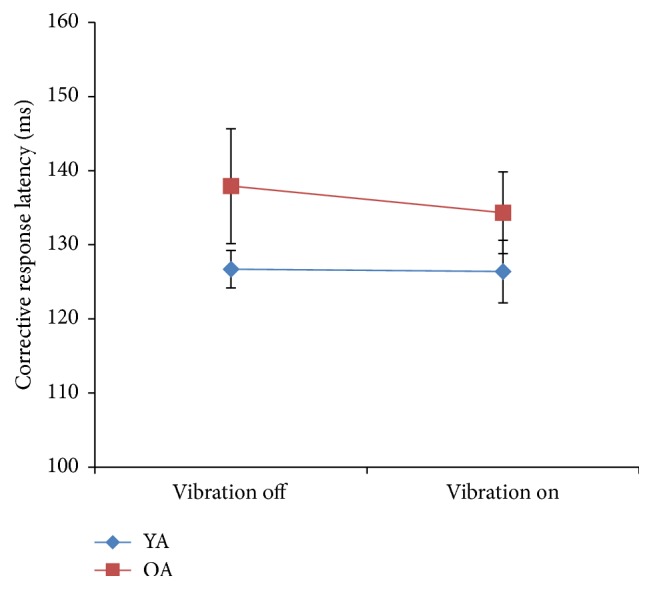
Latency of corrective responses in postural perturbation task between older adults (OA) and younger adults (YA).

Latency of corrective responses (Figure 4) was different between groups, *F*(1,18) = .032 and *η*_*p*_^2^ = .232. There was no significant interaction effect; vibration did not affect latency in older or younger adults.

## 5. Discussion

The current experiment was designed to investigate the differences between older and younger adults when facing postural perturbations. Further, we aimed to evaluate whether a vibration intervention could affect temporal and spatial measures of postural performance and postural control, with the ultimate goal to improve performance when facing balance threats. Participants were instructed to quietly stand on the force plate, which then moved repeatedly in the posterior direction (posterior translation). Repeated trials were performed and spatial and temporal postural measures were analyzed.

### 5.1. Age-Related Associations

Our hypothesis was that older and younger adults' postural performance would differ, which was confirmed for two of the three outcome measures (APPlength and Latency). The effectiveness of neuromuscular responses during support surface perturbations depends on the generation of muscular contractions of the lower body (and trunk) and on the accuracy and adequacy of such responses.

The main goal of the combination of passive and active mechanisms in place for such situations is the avoidance of a fall and the prevention of excessive displacement of the body's COM. Alternatively, humans can react quickly by applying a stepping strategy to maintain balance (increasing the area of the base of support). In our study, participants did not actually generate a stepping response in any of the trials, and therefore balance maintenance was based on a quick response strategy involving generation of torque exerted on the force plate via the feet.

Our results from analysis of maximum COP excursion suggest that age is not necessarily associated with the maximum displacement of COP when facing postural perturbations. The observed similarities between older and younger adults may be due to a number of compensatory mechanisms and strategies. Although it is known that reaction times, muscular strength, and muscular coordination are affected by aging, excursion of COP during perturbations might be controlled in older humans by increasing the magnitude of muscular responses compared to younger adults, to counteract the surface translation. This is possible since the overall joint torques required to maintain balance in quiet stance or when facing perturbations are still generated well by older healthy adults [17]. Alternatively, potential modification of balance strategies supports the compensation of some sensorimotor effects of aging, as indicated by often observed increased agonist-antagonist cocontraction associated with increased lower leg stiffness [51, 52]. Additionally, a reversal of the regular muscle activation order when facing perturbations (from distal-to-proximal strategy to proximal-to-distal strategy) has been reported as another strategy in older adults [53].

Results from perturbation studies in older adults are controversial, since some research has shown that aging does not affect COM measures [54], whereas another evidence suggests that aging increases COM and COP displacements in backward or forward platform perturbation [17, 55, 56]. Results from the current study support findings by de Freitas et al. [54] who found changes in onset of postural musculature EMG activity in older adults when facing postural perturbations and due to aging (starting in the fifth life decade). However, changes in EMG activity due to aging were not reflected in changes of kinematic measures (joint angle excursion for ankle, knee, and hip, maximum displacement of COM, and COM time-to-reversal after onset of perturbation). Those earlier results could indicate that changes in onset of neuromuscular activity are an adaptive strategy that enables older adults to maintain overall postural control strategies similar to those used by younger adults. Results from the current study provide evidence for the ability of healthy older adults to effectively maintain postural balance, similar to younger adults.

The onset of corrective responses was observed primarily within the range of 120–140 ms, whereas group averages in an earlier study were between 155 and 165 ms [50]. This discrepancy could be due to methodological differences between the two experiments. In the former experiment, the amount of translation was fixed, whereas in the current study, translation amplitude was calculated based on each participant's height. Additionally, in the current study, participants knew prior to each trial the perturbation's direction (posterior translation), whereas in Mueller & Redfern's study, 50 consecutive trials of randomized (25 anterior versus 25 posterior translations) order were conducted. Not having accurate knowledge of the direction of the impending perturbation would logically eliminate the ability to implement anticipatory activity that may have influenced response latency. Conversely, the current participants knew what direction the perturbation was to occur and could prepare accordingly. This would, in turn, have an effect of response onsets. It is also possible that, over the duration of 50 perturbation trials, onset of fatigue could have negatively affected latency over time and may have led to results that were different from the ones in the current study.

In the current experiment, groups differed in APPlength, with the older adults displaying more COP total displacement over the course of each trial (2.5 seconds). This result is not in agreement with results from maximum COP excursion analysis. This may indicate a loss of postural stability with aging when facing perturbations of the support platform. Alternatively, it is possible that APPlength in our case is not an indicator of decreased postural performance but an indicator of more exploratory behavior in the older group and as a means of increasing sensory feedback. Sway stimulates muscle spindles and cutaneous receptors of the foot, which in case of the current experiment are important contributors of sensory cues. This sensory information is then used to determine postural orientation and to generate effective responses. Potentially, the younger group required less sway since their sensory feedback operates at near-optimal levels, whereas older adults required this strategy for better performance.

### 5.2. Effects of Subthreshold Vibration

The vibration intervention applied in the current experiment did not have significant effects on any of the analyzed outcome measures. Sensory feedback is considered crucial for the generation of corrective responses (with adequate force production) and the generation of postural adjustments, even before the onset of the perturbation. Relatively strong perturbations as administered in this experiment may not be affected by a fairly subtle improvement of sensory feedback. It is possible that SR effects are more likely to appear (per definition) when otherwise undetectable small magnitude stimuli occur.

However, it is still possible that, over time (e.g., more trials), participants could benefit from enhancements of foot sole feedback to better prepare (e.g., a leaning strategy) for the perturbation and to learn to exploit the enhancement of foot sole feedback.

Alternatively, a reason for lack of observed effects is that the older adults recruited for this study were highly active and exhibited no functional impairments: for gait, mechanical SR stimulation has been shown to be especially effective in more unstable individuals (those exhibiting higher gait variability) when applied to the soles of the feet [57]. Similar results were found for SR based interventions regarding postural sway. In a comparison between stroke patients, diabetic neuropathy patients, and healthy older adults, it was shown that the impact of SR on postural performance improvement depends on initial baseline performance [27]. The authors concluded that it is possible to predict the magnitude of the SR effect by evaluating baseline performance.

Since the effects of SR seem to be dependent on baseline levels of performance, the group recruited for this study could have consisted of “too-high-functioning” individuals. Considering the earlier results, it would be valuable to test the intervention in more frail individuals and those suffering from severe neuropathy. Such investigations of the relationship between severity of postural sensorimotor impairments and effects of the intervention in challenging reactive postural perturbation tasks would provide important information about the value for individuals at the highest risk for falls.

## 6. Conclusion

Healthy aging is associated with some changes regarding specific aspects of control and performance in postural perturbation tasks, whereas healthy older adults are able to compensate for potential functional loss to maintain stability.

The observed discrepancy between different postural outcomes highlights the importance of including a variety of different measures in postural control research. Although a number of different outcome variables may be predictors of falls, they detect and evaluate different components of postural performance and should be in agreement with experimental hypotheses. Analysis of a variety of different measures including spatial and temporal components is valuable to gain further insight into posture and potential effects of aging or interventions.

A low-level vibration intervention seems to not be effective in modifying postural response patterns when facing a support surface perturbation. More research is needed to investigate whether there are benefits for more frail individuals or those suffering from neuropathies.

## Figures and Tables

**Table 1:** Anthropometric characteristics and foot sole vibration threshold of younger and older participants.

	*N*	Gender	Height	Weight	Age	Foot length	% of vib.
Younger	10	f 5	165.6 ± 9.2	148.3 ± 27.2	25.1 ± 2.3	25.4 ± 2.3	2.1 ± 0.6
		m 5					

Older	10	f 8	165.6 ± 10.6	151.1 ± 35.2	78.6 ± 5.4	25.4 ± 1.6	23.2 ± 21.8
		m 2					

**Table 2:** Summary of results from experiment 3. YA: younger adults, OA: older adults (*n* = 20).

	Maximum COP excursion (in cm)		APPlength (in cm)		Latency (in ms)	
	YA	OA	YA	OA	YA	OA
No vibration	5.95 ± 0.6	5.95 ± 1.4	15.77 ± 2.5	21.87 ± 7.8	126 ± 7	138 ± 11
Vibration	5.8 ± 0.9	6.16 ± 1.4	15.0 ± 4.2	20.88 ± 5.5	126 ± 5	134 ± 13
